# An Investigation of Nanomechanical Properties of Materials using Nanoindentation and Artificial Neural Network

**DOI:** 10.1038/s41598-019-49780-z

**Published:** 2019-09-12

**Authors:** Hyuk Lee, Wai Yeong Huen, Vanissorn Vimonsatit, Priyan Mendis

**Affiliations:** 1 Curtin University, School of Civil and Mechanical Engineering, Perth, WA Australia; 2 University of Melbourne, Department of Infrastructure Engineering, Victoria, Australia

**Keywords:** Civil engineering, Mechanical properties

## Abstract

Mechanical properties of materials can be derived from the force-displacement relationship through instrumented indentation tests. Complications arise when establishing the full elastic-plastic stress-strain relationship as the accuracy depends on how the material’s and indenter’s parameters are incorporated. For instance, the effect of the material work-hardening phenomenon such as the pile-up and sink-in effect cannot be accounted for with simplified analytical indentation solutions. Due to this limitation, this paper proposes a new inverse analysis approach based on dimensional functions analysis and artificial neural networks (ANNs). A database of the dimensional functions relating stress and strain parameters of materials has been developed. The database covers a wide range of engineering materials that have the yield strength-to-modulus ratio (*σ*_*y*_/E) between 0.001 to 0.5, the work-hardening power (*n*) between 0–0.5, Poisson’s ratio (*v*) between 0.15–0.45, and the indentation angle (*θ*) between 65–80 degrees. The proposed algorithm enables determining the nanomechanical stress-strain parameters using the indentation force-displacement relationship, and is applicable to any materials that the properties are within the database range. The obtained results are validated with the conventional test results of steel and aluminum samples. To further demonstrate the application of the proposed algorithm, the nanomechanical stress-strain parameters of ordinary Portland cement phases were determined.

## Introduction

Mechanical properties of a wide range of materials that exhibit both elastic and plastic behaviour can be derived from the force-displacement relationship obtained through instrumented indentation^1,2^. Together with the power-law, (*n*) of the work-hardening behaviour, the force-displacement (*P*−*h*) data acquired from the instrumented indentation can be used to derive a full elastic-plastic stress-strain relationship. In recent years, solution algorithms have become increasingly complicated due to the need to incorporate more materials and indenter’s parameters. To overcome this complexity, researchers resorted to finite element (FE) approaches to a wider range of variations in the indentation process^3,4^. One particular research subject that had gained much attention was to determine the effect of the material work-hardening behaviour in the indentation process^5^. It was noted though that the work-hardening phenomenon, such as the pile-up and sink-in effect, could not be accounted for with simplified analytical solutions. Due to this limitation, an investigation of the work-hardening phenomenon is traditionally relying on highly sophisticated imaging technology and equipment^6^. Following the work proposed by Cheng and Cheng^7,8^ dimensional analysis has become viable in developing a relationship between the material work-hardening behaviour and its mechanical properties such as elastic modulus, *E*, and yield strength *σ*_*y*_. The dimensional analysis approach enables using various forward and reverse engineering algorithms for determining the mechanical properties and material work-hardening behaviour^9–11^. Another point to note is that past researches usually focused on determining the main parameters such as the modulus, *E*, and the hardness, *H*, while other parameters such as the Poisson’s ratio, *ν*, and the contact area, *A*_*c*_ at the loading point would usually be kept constant for simplicity^12,13^. This paper extends the Cheng and Cheng’s method of dimensional analysis^8^ to incorporate the effect of the Poisson’s ratio *ν* and the inclination angle *θ* of the indenter’s tip in establishing the stress-strain relationship of the materials. A conventional approach to solving the various parameters presented with the dimensional analysis was usually done by curve fittings whereby the results tend to vary depending on the fitting parameters^9,14^. In this paper, a new approach is proposed by using the dimensional analysis and the Bayesian regularisation training algorithm of the artificial neural networks (ANNs), which does not depend on conventional curve fittings. As the number of unknown parameters increases, the ANNs approach has proven to be practical in finding the parameters’ relationship where it becomes too complicated to relate in a conventional manner.

## Methodology

Inverse analysis algorithms for determining material properties rely on several methods but a significant assumption is to reduce the number of unknowns in the problem. One method is based on the concept of representative stress and strain^9^. Another method which leads to an unknown reduction for dimensional analysis is described by Cheng and Cheng^8^. Although these methods are well established, the effect of the indenter’s geometry and Poisson’s ratio have not been thoroughly considered as they were always kept constant. In this paper, the effect of *ν* and *θ* are incorporated as variables in the dimensional analysis. The indentation result and the derivation of the hardness and the elastic modulus presented in this work is based on the work proposed by Oliver and Pharr^14,15^, which is known as the Oliver-Pharr’s method. The parameters essential for the derivation of the stress-strain characteristics of the material are the unloading stiffness *S*, the reduced elastic modulus *E*_*r*_, and the contact area *A*_*c*_ during the measured loading force *F* and the corresponding displacement *h*. Thus, the identification of the loading and unloading force-displacement from the nanoindentation can be written as follows:

1 $$F=f(E,{\sigma }_{y},\nu ,n,h,{h}_{max},\theta )$$

2 $${\sigma }_{y}=\frac{F}{{h}_{\max }^{2}{\Pi }_{1}}(\frac{{\sigma }_{y}}{E},n,\nu ,\theta )$$

3 $$\begin{array}{rcl}S & = & {\frac{{\rm{d}}F}{{\rm{d}}h}|}_{h={h}_{max}}\\  & = & E{h}_{max}[{h}_{max}{\widehat{\Pi }}_{2}(\frac{{\sigma }_{y}}{E}\mathrm{,1,}n,\nu ,\theta )+2{\widehat{Pi}}_{2}(\frac{{\sigma }_{y}}{E}\mathrm{,1,}n,\nu ,\theta )]\\ \mathrm{or},\,\frac{S}{2E{h}_{max}} & = & {\Pi }_{2}(\frac{{\sigma }_{y}}{E},n,\nu ,\theta )\end{array}$$

4 $$\frac{{W}_{u}}{E{h}_{\max }^{3}}=\frac{{\int }_{0}^{{h}_{\max }}F{\rm{d}}h}{E{h}_{\max }^{3}}={\Pi }_{3}(\frac{{\sigma }_{y}}{E},n,\nu ,\theta )$$

5 $${W}_{t}=\frac{E{h}_{\max }^{3}}{3}={\Pi }_{4}(\frac{{\sigma }_{y}}{E},n,\nu ,\theta )$$

where *θ* is the apex angle of the indenter’s tip, *h* is the indentation displacement corresponding to *F*, and *W*_*u*_ and *W*_*t*_ are the unloading work and the total work derived for the third and fourth dimensional functions, respectively. The four universal dimensional functions, Π_1_, Π_2_, Π_3_, and Π_4_, are the key unknowns for developing the relation between the indentation responses and material properties. In determining Π_1_, Π_2_, Π_3_, and Π_4_, a design of experiment (DOE) approach^16^ is adopted considering four variables viz., *σ*_*y*_/*E*, *n*, *ν*, and *θ* with their ranges are as: *σ*_*y*_/*E* = 0.001 to 0.5, *n* = 0 to 0.5, *ν* = 0.15 to 0.45, and *θ* = 65 to 80 degree. Thus, there are 2496 cases covering the combination of these variables and ranges to be analysed (see more details in the supplementary information). The number of dataset can be reduced by narrowing the *σ*_*y*_/*E* ranges but when dealing with unknown properties of the nanocomponents in the material, it may affect the accuracy of the results. f the parametric range of the target nanomechanical properties is known, the dataset can be reduced. Artificial neural networks (ANNs) have been widely used to predict the nonlinear system due to high precision, low cost and time. The ANNs are computational systems that simulate the neurons of a biological nervous system which is a parallel processing network to determine the complex relationship between input variables and output parameters^17–19^. The architecture of ANNs present the connections between neurons and layers. The connections of weight and biases can be determined using a learning algorithm. ANNs are composed of simple elements operating in parallel, i.e., the simple clustering of the primitive artificial neurons. This clustering occurs by creating layers, which are then connected to one another. General ANNs interfaces are the input-layer and output-layer while all the rest of the neurons are hidden layers. The details of the fundamental description of natural nerves system are available in many textbooks^18,20–22^. The process of Creating ANNs in the present study can be summarised as: involves four main processes: data collection, training of the neural network, validation, and simulation. In the present study, the data for training was 70% of the total data, for validation was 15%, and for simulation was 15%. Further detail of these processes are explained next.

### Data collection

**Figure 1:**
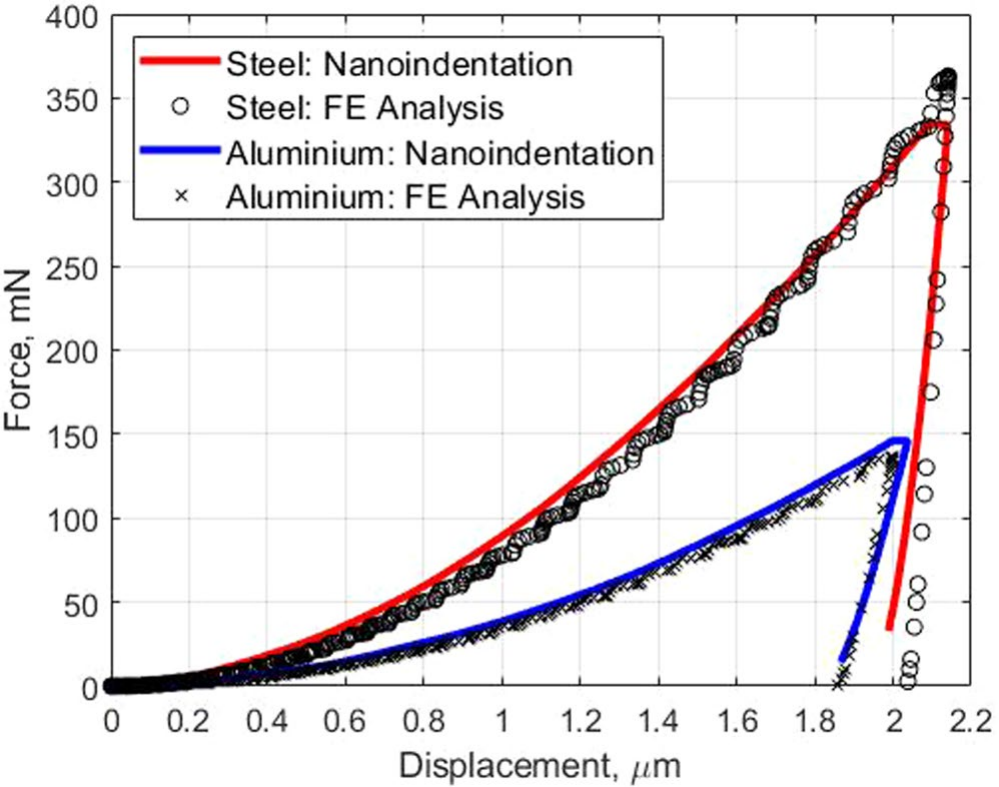
FE analysis and indentation experimental force - displacement for two reference materials.

**Figure 2:**
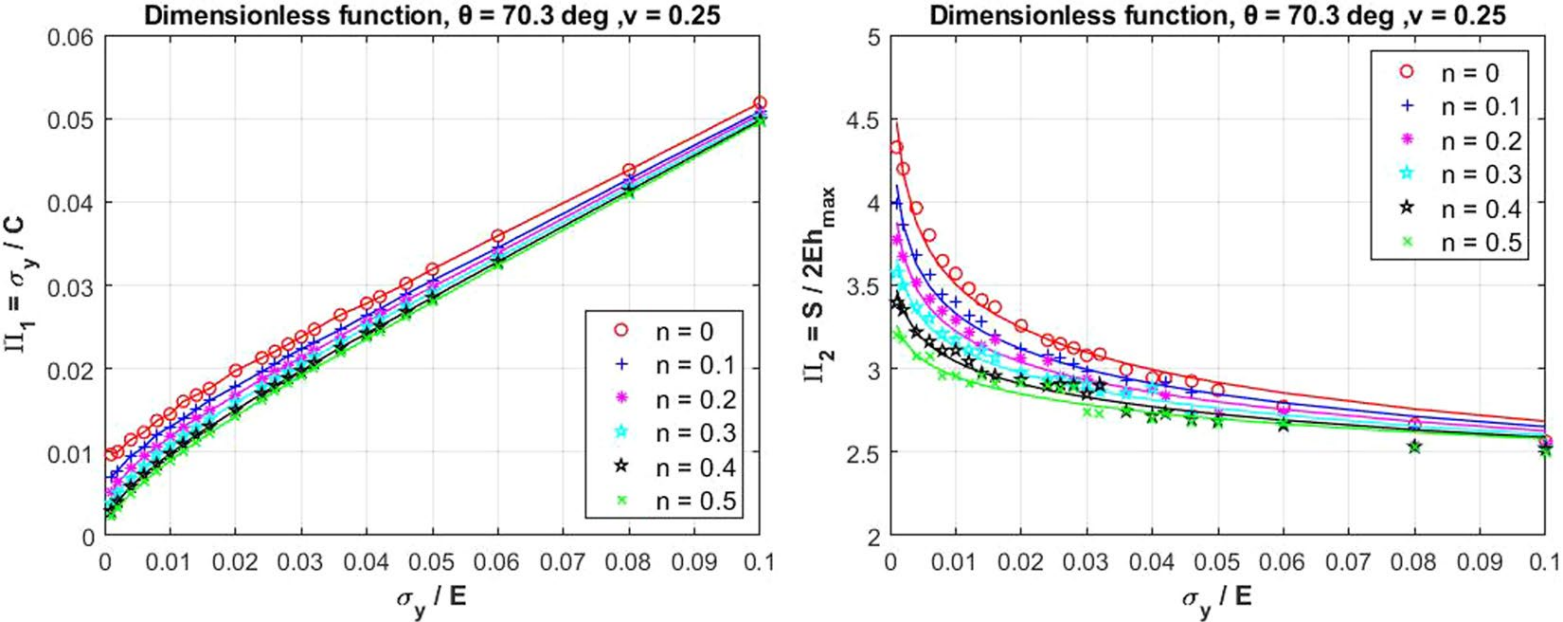
Dimensional functions Π_1_, and Π_2_ for conventional nanoindentation.

**Figure 3:**
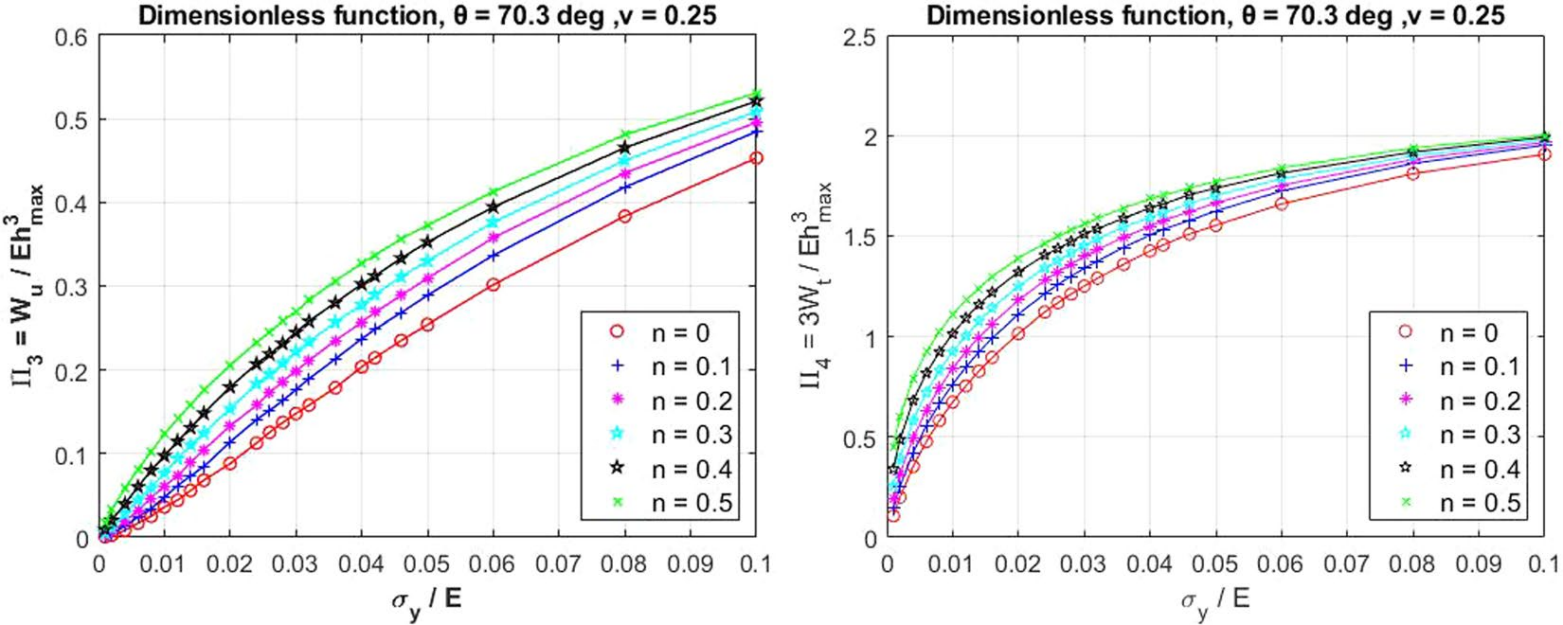
Dimensional functions Π_3_, and Π_4_ for conventional nanoindentation.

Numerical analyses of the material nanoindentation are required for the pre-processing of the input data for the ANNs system. A three-dimensional indenter such as conical or Berkovich indenter can be ideally represented by an axisymmetric two-dimensional model. The conical indenter has the projected contact area, *Ac* = *πh*_*c*_tan^2^*θ*, and the Berkovich indenter has ${A}_{c}=24.56{h}_{c}^{2}{\tan }^{2}\theta$. Therefore, an axisymmetric two-dimensional finite element (FE) model was constructed in ANSYS^23^ to simulate the indentation response of the elastic-plastic behaviour of the material according to this indenter shape. In the FE model, the sample was modelled with 8 or 6 nodes element (PLANE183). The thickness and the radius of the sample were 50 *μ*m, the indentation depth was 2 *μ*m, and the analysis was carried out using a non-symmetric solver. Yurkov *et al*.^24^ investigated the friction of the diamond indenter’s tip on various material contact surfaces such as steel, sapphire, alumina and fused silica. They concluded that the friction coefficient depends on the indentation load. To account for this effect, the friction between the indenter and the sample was chosen to be 0.15 in the numerical analysis. To verify the FE analysis, two reference materials, steel and aluminium alloy, were tested and compared with the indentation experiments. Three 12 mm bars of each reference material were tested according to ASTM E8M^25^. Nanoindentation test was also carried out on the two reference samples using XP (all purpose) testing model with the applied displacement of 2 *μ*m. Berkovich indenter (equivalent conical angle = 70.3°) was used for the entire testing and the samples were prepared according to ASTM E3^26^. As shown in Fig. 1, the results from the nanoindentation test and from the numerical analysis agree very well. The slight difference between the numerical and experimental results could be due to the effect of the surface roughness of the samples, as also pointed out in the literature^27,28^. The FE results of the dimensional functions from all the combination of the parameters as outlined in the DOE table in the supplementary information are then obtained. Figures 2 and 3 show a typical series of the relationships between various *σ*_*y*_/*E* ratios and the dimensional functions,Π_1_, Π_2_, Π_3_, and Π_4_, with varying *n* values. Due to a space limitation, only the cases with *ν* = 0.25 and *θ* = 70.3 degree are presented in this paper. Conventionally, a curve fitting method can be used to determine specific parameters from these curves. However in this case, while the FE results contain a non-linear relationship of the dimensional functions with varying *ν* and *θ*, deriving the parameters using the curve fitting method by force fitting the relationships of the three dimensional parameters would result in a loss of the accuracy due to this simplification. Not to mention that the three-dimensional curve fitting is practically difficult to perform manually. Therefore, this paper proposes using the artificial neural network (ANNs) as an alternative method for finding the parameters based on the relationship derived from the FE analysis.

### Training of the neural network

**Figure 4:**
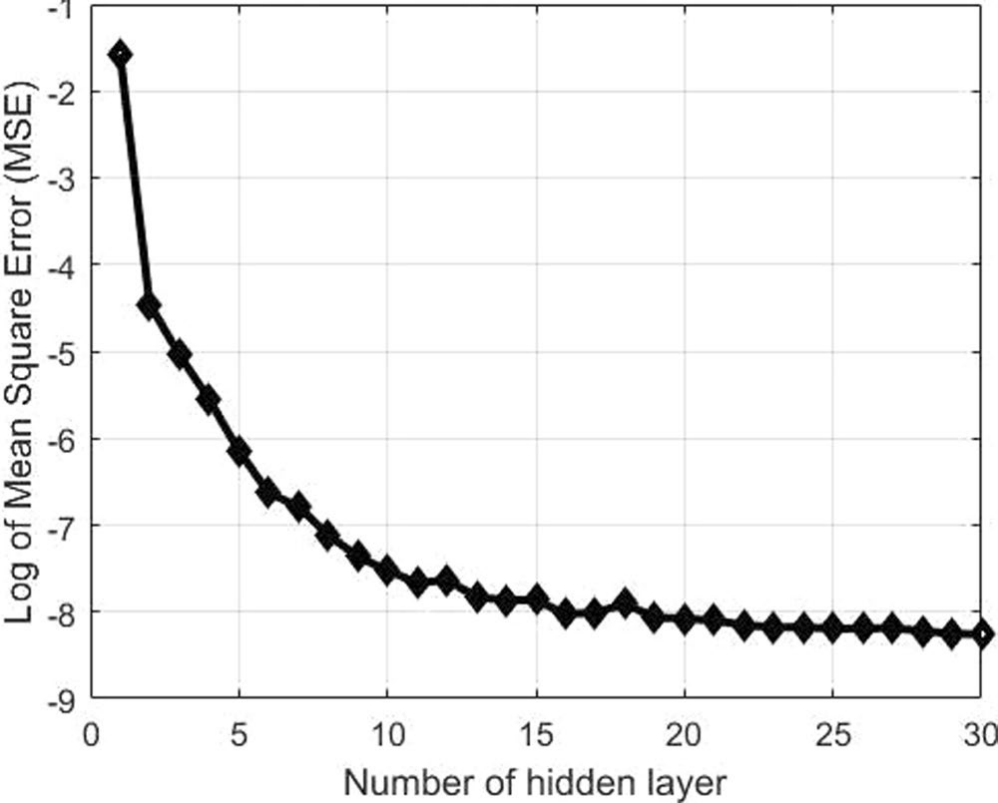
Convergence of hidden layer in ANNs.

For the training of the neural networks, it is required to choose the number of hidden layers. The optimal network configuration has been chosen with 30 hidden layers from the convergence with Mean Square Error (MSE) as shown in Fig. 4. The hidden layers of the neural network have been considered as tangent-sigmoid. The quantitative evaluation of the diagnostic performance of the ANNs for the dimensional functions was accomplished by performing 10,000 iterations of the training procedure with the gradient of 1.00e−7 and the standard deviation of 0.005.

### Validation and simulations of the neural network

**Figure 5:**
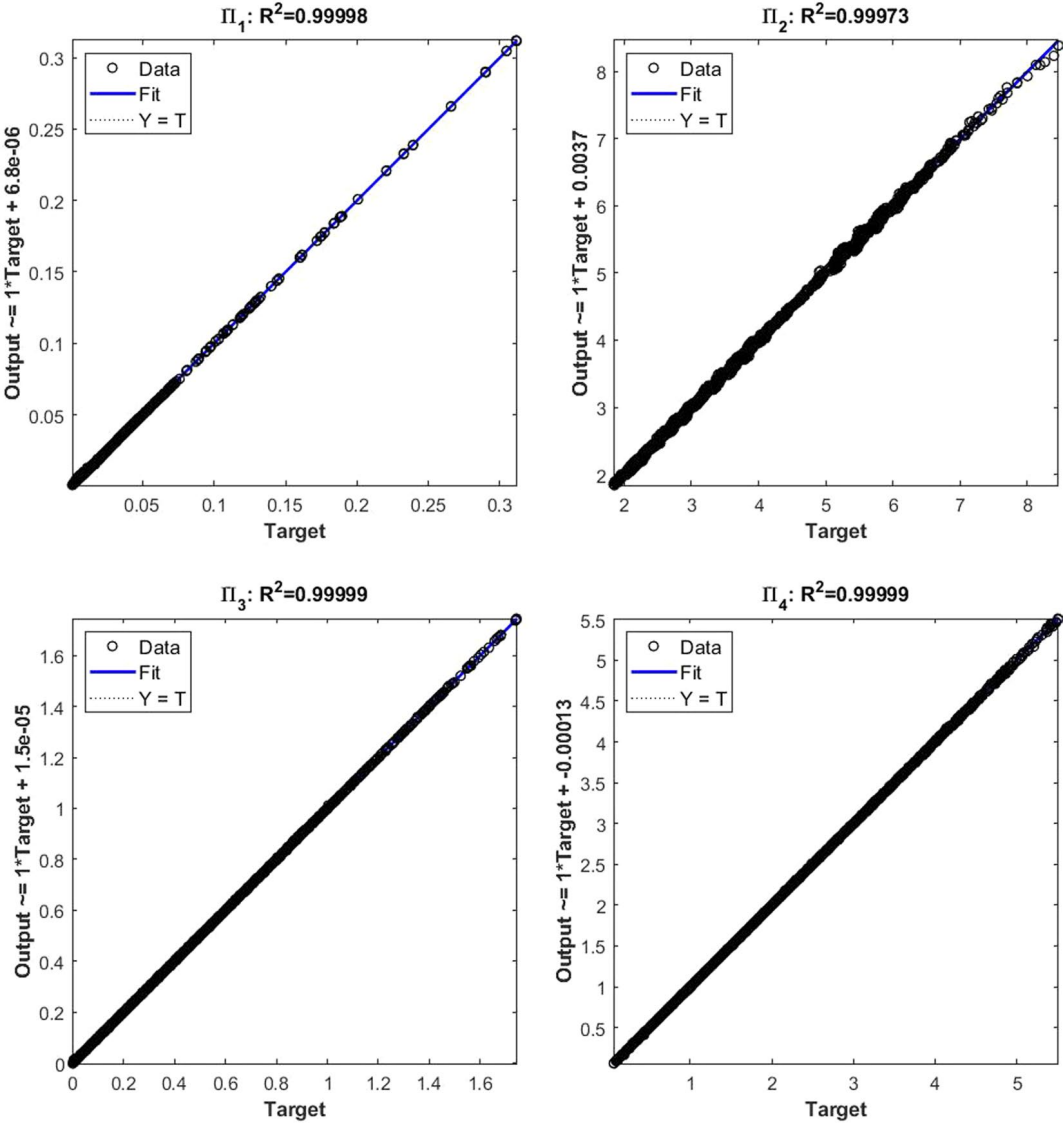
Correction coefficient of ANNs with 30 hidden layers.

The quality of the prediction is characterised by the root mean square error (RMSE) of the predicted values from the input data. Similarly, the correction coefficient *R*^2^ has been used to evaluate the ANNs’ quality. The results of the empirical correction of the neural network training show a good agreement with the target values, as shown in Fig. 5. The output of the dimensional functions can be expressed as:

6 $$\Pi ={B}_{2}+LW\cdot tansig({B}_{1}+IW\cdot x)$$

where *x* is the input parameters from the four dimensional functions (4 × 1), *B*_1_ is a bias vector (30 × 1), i.e., for 30 hidden layers and biases, and *B*_2_ is a scalar. *LW* is the weight matrix (1 × 30) between layers, and *IW* is the matrix (30 × 4) of the weights going to the hidden layers from the network input. Thus, the dimensional functions have been established with ANNs from the four input parameters. A comprehensive parametric study of the 2496 cases was then conducted to represent the range of the parameters of the mechanical responses found in the ANNs.

## Inverse Algorithm

**Figure 6:**
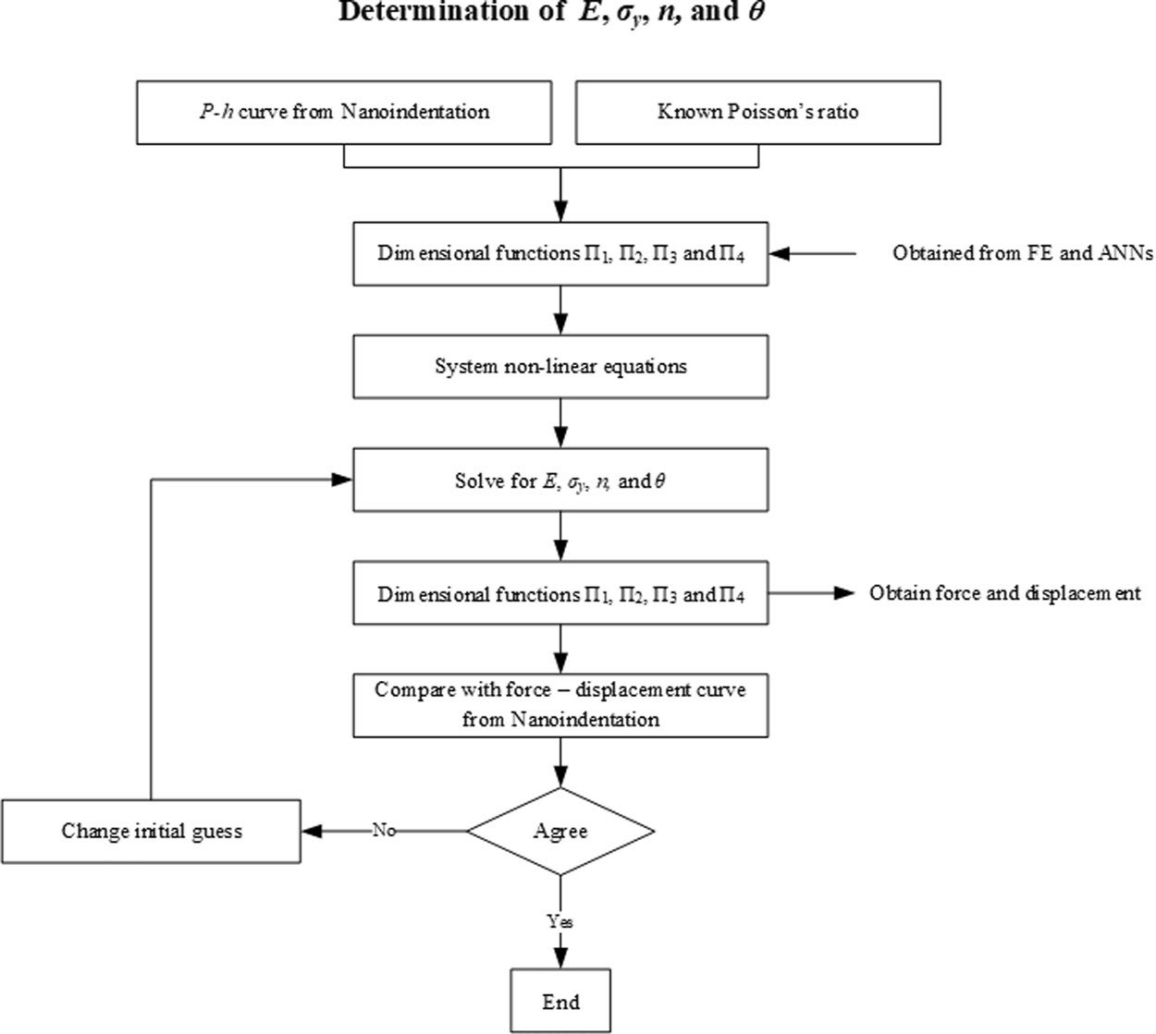
Flow chart: Inverse analysis algorithm.

Figure 6 shows the flowchart of the inverse analysis algorithm used in this study for determining the stress-strain characteristics of the materials. The step-by-step procedures are as follows: (i) carry out an indentation experiment, with the known *ν* of the sample, to obtain the indentation force – displacement (*P*−*h*) curve, (ii) determine the key parameters of the *P*−*h* curve, which are *F*, *h*_*max*_, *S*, *W*_*u*_ and *W*_*t*_, (iii) solve the system equations, Eqs (1) to (5), using a nonlinear system solver according to the values of the dimensional functions obtained from the trained ANNs, to determine the unknowns *E*, *σ*_*y*_, *n*, and *θ*, and (iv) validate the values of *F*, *h*_*max*_, *S*, *W*_*u*_ and *W*_*t*_. The process is complete when the reproduced *P*−*h* curve matches the tested curve. The obtained *E*, *σ*_*y*_, *n*, and *θ* values are specific to the indentation location as other locations may have a different *P*−*h* curve due to the material’s inhomogeneity and anisotropic behaviour. To solve the non-linear equations, a reasonable initial guess is required to predict the solution. For example, an initial *E* can be assumed based on the Oliver and Pharr approach using *σ*_*y*_ = 0.001, *n* = 0.05 and *θ* = 70.3, which are a reasonable initial guess for solving the non-linear equations. If the validation results do not agree with the experimental results, the next step is to change the initial guess values of the yield strength to elastic modulus ratio and the power law work hardening.

**Figure 7:**
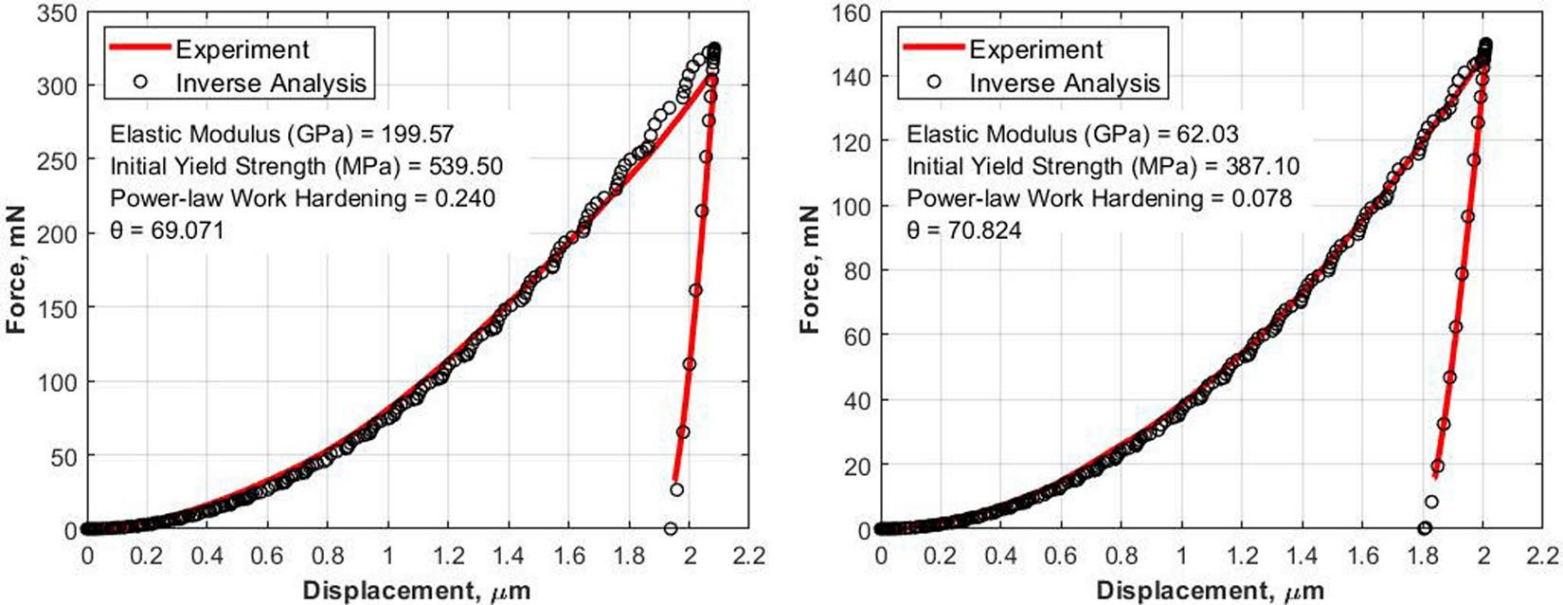
Force-Displacement data comparison between experiment and inverse analysis steel(left) and aluminium(right).

To verify the inverse algorithm, two reference samples, steel and aluminum were studied. The force-displacement results obtained from the indentation experiment and the proposed inverse analysis are as shown in Fig. 7. It is clear that the proposed algorithm provides an accurate estimate of *E*, *σ*_*y*_, *n*, and *θ* that agree well with the experimental results. Errors have been intentionally imposed on the experimental data to test the sensitivity of the inverse algorithm to the errors. It should be noted that the value of the angle *θ* (in this case, 69.1 for steel and 70.8 for aluminum) obtained from the inverse analysis is different from the ideal equivalent cone angle of the Berkovich indenter (*θ* = 70.3 degrees). This difference can play an important role in the determination of the mechanical properties from the inverse algorithm. The specimen’s model in the FE analysis is usually assumed to have a perfect surface treatment, but due to the limitation of the cutting tool and mechanical dynamic, it is difficult to get the perfect surface treatment from the machining operation. According to the literature^2,28–30^, the loading curves are affected by the surface roughness, while unloading curves are not significantly affected. That means, the difference in the *θ* value from the inverse algorithm could be due to the surface roughness of the tested samples. The effect of the surface roughness is due to the influence of the contact area which affects the equivalent cone angle *θ*. For example, a reduction in the contact area leads to a decrease in the equivalent cone angle, while an increase in the contact area leads to an increase in the equivalent cone angle.

## Application

**Figure 8:**
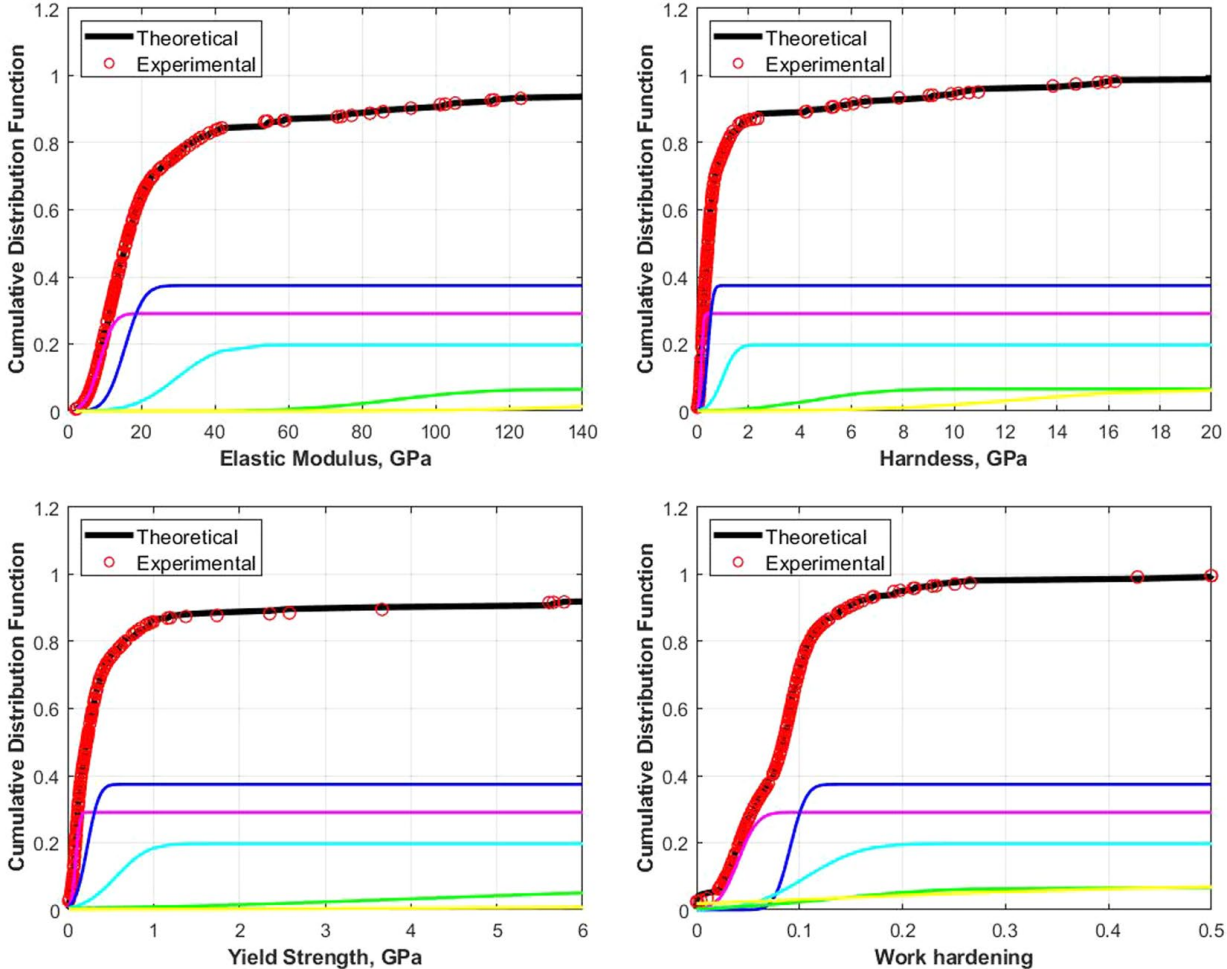
Deconvolution results of OPC paste.

An application of the proposed inverse algorithm is demonstrated in this section. OPC paste samples were made of General purpose (Type I) cement available locally in Australia. Five sets were made, all with the water to cement ratio of 0.3 and cured in lime water at ambient temperature 23 ± 3 °C after casting. A compressive strength test carried out according to ASTM C109M^31^ provided the average strength of 94.6 MPa at 28 days curing age. For the Nanoindentation test, three samples were taken from each of the five sets of the OPC paste. A minimum of 10 × 10 grid indentation was set per each sample. The hardness (*H* = *F*/*A*_*c*_), *E*, *σ*_*y*_ and *n* values were then determined using the inverse algorithm. The indenter’s angle was a contributing unknown, thus, the inverse analysis could be used to further investigate the effect of the surface roughness and the contact areas of the tested samples. The hydration products of OPC paste contain five major phases, which are capillary porosity, low-density calcium silicate hydrated (LD CSH), high calcium silicate hydrate (HD CSH), Portlandite (CH) and clinker with calcium silicate hydrated (CSH)^32,33^. It has been evident^32–35^ that calcium silicate hydrated (CSH) is a governing component contributing to its fundamental properties, such as strength, relaxation, creep and fracture behaviours. Understanding CSH characteristics and how they relate to other local mechanical properties requires a mechanical characterisation of the nanostructure of OPC pastes. The proposed inverse algorithm provides nanomechanical properties such as yield strength and power law work hardening of the major phases in the hydrated OPC. Due to the heterogeneity nature of the OPC pastes, a deconvolution technique was used to determine the indentation properties of the hydration phases. The results are illustrated in Fig. 8. The values of *E*, *H*,*σ*_*y*_ and *n* and the percentage of the volume fraction of each phase are presented in Table 1.

**Table 1:** Deconvolution results of OPC paste.

Phase	*E* (GPa)		*H* (GPa)		*σ*_*y*_ (MPa)		*n* (GPa)		Volume fraction%
	mean	StD	mean	StD	mean	StD	StD	mean	
MP	8.695	3.128	0.187	0.076	0.093	0.039	0.040	0.015	28
LD CSH	15.521	4.115	0.442	0.153	0.229	0.110	0.091	0.012	37
HD CSH	29.651	9.224	1.010	0.415	0.565	0.293	0.107	0.042	20
CH	88.231	19.834	4.592	2.083	4.017	2.702	0.133	0.085	8
Clinker	180.164	43.928	12.969	4.627	16.145	8.040	0.148	0.226	7

The average values of the first peak shows *E* = 8.695 GPa, *H* = 0.187 GPa, *σ*_*y*_ = 0.093 GPa, and *n* = 0.040; the second peak, *E* = 15.521 GPa, *H* = 0.442 GPa, *σ*_*y*_ = 0.229 GPa, and *n* = 0.091; the third peak, *E* = 29.651 GPa, *H* = 1.010 GPa, *σ*_*y*_ = 0.565 GPa, and *n* = 0.107. For the fourth peak, *E* = 88.231 GPa, *H* = 4.592 GPa, *σ*_*y*_ = 4.017 GPa, *n* = 0.133, and the clinker, *E* = 180.164 GPa, *H* = 12.969 GPa, *σ*_*y*_ = 16.145 GPa, *n* = 0.148. The results are within the range of the *E* and *H* values reported in literatures depending on the mixture compositions^32–34^, i.e., the *E* value of LD CSH is 12 to 22.2 GPa, of HD CSH is 25.8 to 33.1 GPa, and the *H* value of LD CSH is 0.5 to 0.6 GPa, and of HD CSH is 1.2 to 1.0 GPa. The results of deconvolution with inverse algorithm can be provided the analytical stress-strain behaviour of OPC paste. The present results forms a basis towards an on-going development of a scale relation model of the elastic-plastic behaviour of composite materials.

## Conclusion

An inverse algorithm suitable for determining the nanomechanical properties of materials was presented in this paper. The algorithm is based on a combination of a dimensional analysis approach, FE analysis and artificial neural networks (ANNs). The novelty of the present method is in the incorporation of the Poisson’s ratio and the indenter’s angle, which is influenced by the sample’s surface roughness during the indentation, in the dimensional functions. The proposed inverse algorithm enables establishing the elastic-plastic stress-strain relationship of the local phases in the tested material. Based on the experiments and analyses carried out in the present work, the following conclusions can be drawn:

- Artificial neural networks (ANNs) are demonstrated to be an effective tool in determining nanomechanical properties of materials through dimensional analysis. Conventionally the number of unknown parameters in a curve fitting approach is three, which are elastic modulus, yield strength and work hardening power. With the ANN approach, it was demonstrated in this paper that the solution is achievable with the additional two unknowns, Poisson’s ratio *ν* and the indenter (cone) angle *θ*.
- The proposed inverse analysis algorithm can be used to predict the nanomechanical stress-strain parameters of any materials that have *σ*_*y*_/*E* = 0.001 to 0.5, *n* = 0 to 0.5, *ν* = 0.15 to 0.45, and *θ* = 65 to 80 degree.
- The derived parameters obtained from the inverse analysis of steel and aluminum samples show that the determined *E*, *σ*_*y*_, *n*, and *θ* can be validated with the experimental force-displacement relationship.
- The nanomechanical properties of OPC phases were determined; the indentation modulus and hardness agreed well with the values previously published in the literature. The incorporation of the effect of the surface roughness and the material’s Poisson’s ratio in the determination of the yield strength, elastic modulus and work-hardening power of the local properties of the material was successfully achieved.

The present work forms a basis towards an on-going development of a multiscale-link model of the elastic-plastic behaviour of multiphase materials such as cementitious composites.

## Supplementary information

Supplementary Information
