# Supplementary material for: An Investigation of Nanomechanical Properties of Materials using Nanoindentation and Artificial Neural Network
Source: Sci Rep. 2019 Sep 12;9:13189. doi: 10.1038/s41598-019-49780-z (PMC6742636; doi:10.1038/s41598-019-49780-z)
Supplement: Supplementary file 1 — Supplementary Information [file 41598_2019_49780_MOESM1_ESM.pdf]

An Investigation of Nanomechanical Properties of Materials using Nanoindentation and Artificial Neural Network

Hyuk Lee[1], Wai Yeong Huen[1], Vanissorn Vimonsatit[1], and Priyan Mendis[2]

[1] School of Civil and Mechanical Engineering, Curtin University, WA, Australia
[2] Department of Infrastructure Engineering, University of Melbourne, Victoria, Australia

Supplementary Note: Design of Experimental

Based on a Design of Experiment (DOE) approach, the levels of the parameters used for determining the dimensional functions are listed in Table A. In total, 2496 combinations were analysed.

Table A. Parameters and Levels

Levels	σ_y/E	n	v	θ *(degree)*
**1**	0.001	0	0.15	65
**2**	0.002	0.1	0.25	70.3
**3**	0.004	0.2	0.35	75
**4**	0.006	0.3	0.45	80
**5**	0.008	0.4		
**6**	0.010	0.5		
**7**	0.012			
**8**	0.014			
**9**	0.016			
**10**	0.020			
**11**	0.024			
**12**	0.026			
**13**	0.028			
**14**	0.030			
**15**	0.032			
**16**	0.036			
**17**	0.040			
**18**	0.042			
**19**	0.046			
**20**	0.050			
**21**	0.060			
**22**	0.080			
**23**	0.100			
**24**	0.200			
**25**	0.300			
**26**	0.500			
